# Significance of GSH and H_2_S regulation for cancer: an intricate interplay between diet, microbiota, metabolic reprogramming, and immune health

**DOI:** 10.1080/13510002.2026.2687238

**Published:** 2026-06-11

**Authors:** Avisek Majumder, Suravi Majumder, Shabana Bano, Koushik Sen, Kasturi Bala Nayak

**Affiliations:** a Department of Medicine, University of California, San Francisco, CA, USA; b Department of Pharmacological & Pharmaceutical Sciences, University of Houston, Houston, TX, USA; c Department of Zoology, Jhargram Raj College, Jhargram, West Bengal, India; d Department of Medicine, Quantitative Biosciences Institute, University of California, San Francisco, CA, USA

**Keywords:** Persulfide, ferroptosis, lipid peroxidation, methionine restriction, immunotherapy, targeted therapy, oxidative stress, gut microbiome

## Abstract

Due to the proliferative nature of cancer cells, they utilize more dietary extracellular nutrients via one-carbon metabolism for the various metabolic processes, including the synthesis of antioxidants such as glutathione (GSH) and hydrogen sulfide (H_2_S). Indeed, several studies have found that specific cancer types produce significantly higher levels of GSH and H_2_S than normal healthy cells, which may serve as a protective mechanism, allowing them to resist stress, survive, and grow. This metabolic heterogeneity, driven by intrinsic and extrinsic factors, contributes to the distinct metabolic characteristics and vulnerabilities of tumor subtypes, which can be exploited to develop anticancer strategies. In this review, we summarize the fundamental roles and regulation of GSH and H_2_S in normal physiological systems and in the genesis and progression of cancer, their effects on the tumor microenvironment (TME), and their contribution to drug resistance. We also discuss the influences of diet and the gut microbiome on GSH and H_2_S production, and how cancer cells reprogram their metabolism to grow and survive in a stressful environment by overproducing GSH and H_2_S.

## Graphical abstract

**Figure:**
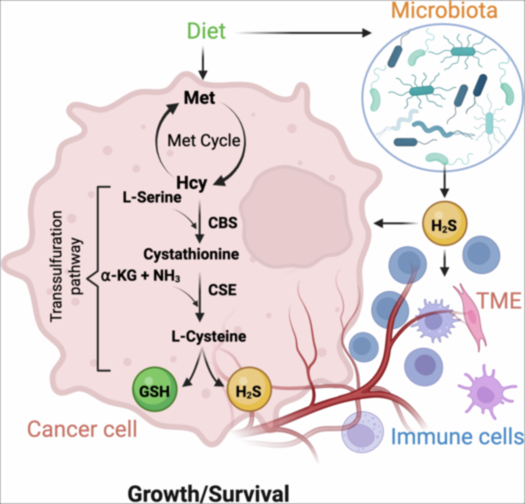

## 1. Background

In 2019, the World Health Organization (WHO) identified cancer as the first leading cause of death in developed countries and the second leading cause in developing nations [1]. The third Expert Report from the World Cancer Research Fund (WCRF) emphasizes that diet and nutrition are critical modifiable risk factors for cancer and its management [2,3]. Focusing on improving dietary choices and nutritional habits can significantly reduce the incidence of various types of cancers, improve treatment outcomes, and promote overall health.

Methionine (an essential amino acid) is the only precursor of one-carbon metabolism that is not produced in our body; it must come from the diet (mainly from animal products) [4]. A diet that is rich in methionine is linked to a higher risk of cancer [5]. Methionine is especially important because it is not only the first amino acid added during protein synthesis but also provides the methyl group (X-CH3) involved in all one-carbon transfer reactions (during DNA, RNA, and protein methylation) in mammalian cells [5]. In addition to its vital roles in methylation reactions and protein synthesis, methionine is essential for the production of key cellular antioxidants: glutathione (GSH) and hydrogen sulfide (H_2_S), thereby helping to maintain redox balance and associated cellular processes [6]. GSH and H_2_S are the primary non-enzymatic antioxidants in the body, and, notably, these metabolites share a common production pathway (i.e. the transsulfuration pathway) and serve similar functions (i.e. reducing oxidative stress). Because of the crucial roles of H_2_S and GSH in cellular homeostasis, the demand for their precursors (mainly methionine and cysteine) is high in cancer cells [4]. While dietary restriction of specific extracellular nutrients may be a less toxic alternative to traditional cancer treatments, immune cells also require these nutrients for survival and growth, further complicating the effectiveness of these treatments [7].

GSH and H_2_S play critical roles as primary intracellular antioxidants, facilitate the generation of reducing equivalents, and effectively prevent the accumulation of reactive oxygen species (ROS) and reactive nitrogen species (RNS) [8]. Both GSH and H_2_S can be made endogenously via the transsulfuration pathway of the methionine cycle. GSH is produced in the cytoplasm and is tightly regulated by substrates, including glutamate, cysteine, and glycine, as well as by essential enzymes such as glutamate-cysteine ligase (GCL) and glutathione synthase (GS) [9]. When GSH regulation is disrupted or altered, it can contribute to cancer initiation, progression, and drug resistance in various types of cancer [10]. H_2_S can be synthesized endogenously primarily via three enzymes: cystathionine-β-synthase (CBS), cystathionine-γ-lyase (CTH), and 3-mercaptopyruvate sulfurtransferase (3MST) [11]. H_2_S can also be produced via non-enzymatic pathways (from elemental sulfur/polysulfides) and through the gut microbiota, forming a total cellular H_2_S pool [12]. As our body cells and gut microbes both produce H_2_S by metabolizing dietary sulfur amino acids, such as methionine and cysteine, it is now well documented that diets that induce sulfur-related microbes in the gut correlate with an increased risk of early-onset colon adenomas and colon cancer (20–22); however, this rationale is poorly studied in other types of cancer. Given that the gut microbiota generates significantly greater amounts of H_2_S than the body cells do endogenously, the origin of H_2_S, whether from bacterial sources or our body cells, becomes a key factor in understanding its impact on cancer.

Hence, to develop novel anticancer strategies, it is important to understand the fundamental roles and regulation of GSH and H_2_S in normal physiological systems, and how their levels are modulated during cancer initiation and progression, as we discussed in this review. The high production of ROS/RNS in cancer cells necessitates increased cellular antioxidant levels (e.g. GSH and H_2_S) for survival. In this review, we also highlighted how cancer cells modulate their cellular GSH and H_2_S production via metabolic reprogramming and the role of diet in this process; how these metabolites modulate the TME; and how high levels of GSH and H_2_S help cancer cells escape therapeutic challenges.

## 2. Production of GSH and H_2_S in the mammalian system

In order to understand the roles of GSH and H_2_S in cancer risk and progression, we need to know how these metabolites are produced and metabolized in the mammalian system. The levels of GSH and H_2_S production can be influenced by dietary factors such as methionine, cysteine, glycine, glutamate, vitamins B12 and B6. In this section, we explain how intracellular GSH and H_2_S pools are generated and metabolized in mammalian systems via multiple mechanisms.

### 2.1. GSH synthesis, recycling, and degradation

**Figure 1.**
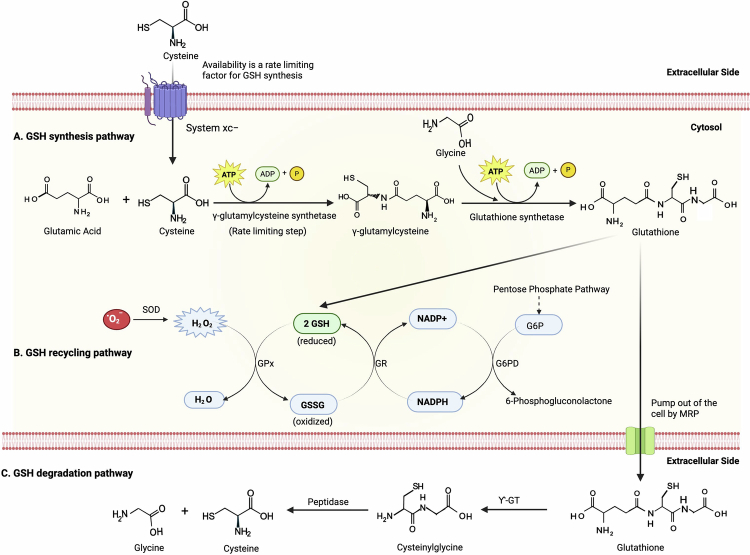
A diagram illustrates glutathione metabolism through synthesis, recycling, and degradation pathways across cell membranes. Glutathione synthesis, recycling, and degradation pathways: Glutathione (GSH) metabolism involves synthesis from glutamate, cysteine, and glycine via γ-glutamylcysteine synthetase (GCS) and glutathione synthetase (GS) (a), recycling from oxidized glutathione (GSSG) by glutathione reductase (GR) (b), and degradation by γ-glutamyl transpeptidase (γ-GT) and other peptidases (c). GSH synthesis maintains cellular antioxidant balance via glutathione peroxidase (GPx), with cysteine availability often limiting GSH synthesis. Abbreviations: SOD, superoxide dismutase; NADPH, nicotinamide adenine dinucleotide phosphate; G6P, glucose-6-phosphate; H_2_O_2_, hydrogen peroxide. This Figure was created using BioRender.

GSH biosynthesis is a highly conserved pathway in which three precursor amino acids—cysteine, glutamate, and glycine—combine to form the tripeptide GSH [9], as illustrated in Figure 1A. This process occurs exclusively in the cytosol, where two key enzymes, GCL and GS, are localized. GCL catalyzes the first, rate-limiting step of the pathway, linking glutamate and cysteine via the γ-carboxyl residue of cysteine to produce γ-glutamylcysteine [9]. This reaction requires ATP hydrolysis and the magnesium (Mg^2+^) as a cofactor. The GCL enzyme comprises two subunits: a catalytic subunit (glutamate-cysteine ligase catalytic: GCLC) and a modifier subunit (glutamate-cysteine ligase modifier: GCLM) [9]. The GCLM subunit is subject to feedback inhibition by high GSH levels [9]. The second and final step of GSH biosynthesis involves the addition of glycine to the γ-glutamylcysteine intermediate, forming GSH [9].

In parallel with GSH biosynthesis, GSH recycling (Figure 1B) and degradation (Figure 1C) pathways are also active in our body [13]. Following synthesis, two reduced forms of GSH molecules are converted to one oxidized glutathione (GSSG) molecule via the formation of a disulfide bond, which in turn neutralizes free radicals. Depending upon the specific ROS substrate, this reaction is catalyzed by specific glutathione peroxidase (GPX) isoforms [4]. GSSG subsequently reduced to regenerate functional GSH monomers by glutathione reductase (GR) and nicotinamide adenine dinucleotide phosphate (NADPH) as a cofactor. Cellular NADPH demand can be replenished via different metabolic pathways, including the oxidative pentose phosphate pathway (as shown in Figure 1B).

GSH is relatively stable because of its unique amide bond formed from a γ-carboxyl group instead of an *α*-carboxyl group observed in most other peptides [13]. However, once GSH is pumped out of the cell by the multidrug resistance-associated proteins (MRPs), its degradation begins by γ-glutamyl cyclotransferase (γ-GT, also known as GGCT or GGT) [14]. This enzyme is exclusively located on the plasma membrane, with its active site facing towards the extracellular space. In the extracellular space, γ-GT hydrolyzes GSH to produce the intermediates cysteinylglycine and 5-oxoproline [14]. Cysteinylglycine is then broken down into cysteine and glycine by its respective peptidase, while 5-oxoproline is converted to glutamate through the action of a 5-oxoprolinase enzyme, which also requires ATP hydrolysis [14].

### 2.2. Different routes of H_2_S synthesis in the mammalian system

H_2_S production is primarily mediated by three processes: endogenous enzymatic pathways, non-enzymatic pathways, and the gut microbiota. The next part provides a detailed description of each pathway.

#### 2.2.1. Enzymatic pathway of H_2_S synthesis

**Figure 2.**
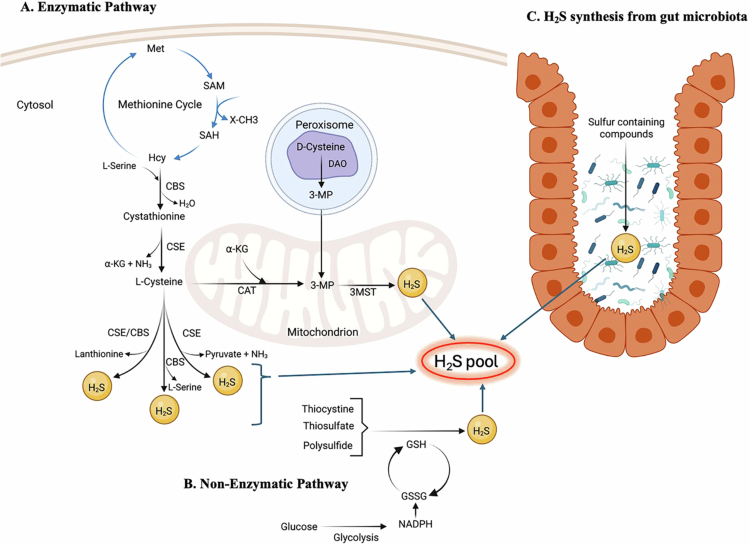
Three panel diagram of hydrogen sulfide synthesis. Panel A: enzymatic. Panel B: non-enzymatic. Panel C: gut microbiota. H_2_S synthesis from endogenous enzymatic pathways and gut microbiota: Hydrogen sulfide (H_2_S) can be synthesized in the body via enzymatic pathways (by CBS, CSE, and 3MST) from sulfur-containing amino acids (a). H_2_S can also be synthesized via non-enzymatic pathways (from elemental sulfur/polysulfides using reductants such as NADPH/GSH) (b) and by the gut microbiota (bacterial breakdown of cysteine and reduction of sulfates) (c). The microbiota significantly influences host H₂S levels, affecting systemic H₂S bioavailability and impacting different cellular processes. Abbreviations: SAM, S-adenosylmethionine; SAH, S-adenosylhomocysteine; Met, methionine; Hcy, homocysteine; α-KG, α-ketoglutarate; CBS, cystathionine-β-synthase; CSE, cystathionine γ-lyase; 3MST, 3-mercaptopyruvate sulfurtransferase; CAT, cysteine aminotransferase; DAO, D-amino acid oxidase; 3MP, 3-mercaptopyruvate. The Figure was created using BioRender.

In mammalian cells, H_2_S is synthesized via an enzymatic pathway primarily through the action of the enzymes CBS and CTH. These enzymes require pyridoxal-5′-phosphate (PLP) as a cofactor and use L-cysteine as a substrate to produce H_2_S [11]. In addition to these well-established pathways, several alternative routes for H_2_S production have also been identified. One notable enzyme in this context is 3MST, which functions independently of PLP [15]. First, cysteine aminotransferase (CAT) catalyzes the production of 3-mercaptopyruvate (3MP) and glutamate from α-ketoglutarate (α-KG) and L-cysteine, as shown in Figure 2A. Subsequently, 3MST catalyzes the transformation of 3MP into pyruvate and H_2_S, with the aid of reducing agents such as thioredoxin or dihydrolipoic acid. Furthermore, in 2013, Shibuya et al. provided important insights by describing another pathway for endogenous H_2_S production [16]. In this pathway, peroxisomal enzyme D-amino acid oxidase (DAO) converts D-cysteine to 3MP, which then serves as a substrate for 3MST in the mitochondria, producing H_2_S.

#### 2.2.2. Non-enzymatic pathway of H_2_S synthesis

Non-enzymatic H_2_S production occurs through several key compounds, including thiosulfate, GSH, glucose, inorganic sulfur, and organic polysulfides (Figure 2B) [12]. Thiosulfate is an important intermediate in sulfur metabolism, generating H_2_S via a reductive reaction with pyruvate, which effectively serves as a hydrogen donor. Moreover, H_2_S can arise from glucose via either the phosphogluconate pathway, which involves NADPH oxidase, or glycolysis [17]. In this context, glucose interacts with amino acids such as cysteine, methionine, and homocysteine, thereby generating gaseous sulfur compounds, including H_2_S and methanethiol [12]. Additionally, H_2_S is produced through the direct reduction of GSH and inorganic sulfur [18]. The reactivity of organic polysulfides is noteworthy; they can undergo nucleophilic substitution at sulfur, yielding H_2_S and hydropolysulfide.

#### 2.2.3. H_2_S synthesis by gut microbiota

Microbes in the gastrointestinal tract play a crucial role in H_2_S production in mammals, with most occurring in the colon (Figure 2C). Luminal H_2_S concentrations are estimated to range from 0.3 to 3.4 mM [19]. Although measuring serum H_2_S concentration in healthy individuals can be challenging, estimates indicate that it typically ranges from 34.0 to 36.4 μM [20]. Systemic H_2_S can induce the total cellular H_2_S pool across different organs and influence physiological processes in other organs.

In the human gut microbiota, H_2_S is produced primarily by two key pathways: dissimilatory sulfate reduction (DSR) and the degradation of sulfur-containing amino acids (e.g. cysteine and methionine) [21]. The DSR pathway is carried out by sulfate-reducing bacteria (SRB), like *Desulfovibrio*, *Desulfobacter*, *Desulfobulbus*, and *Desulfotomaculum*, which reduce various compounds (including sulfate, sulfite, thiosulfate, elemental sulfur, and several thionates) and produce H_2_S [22,23]. Whereas, degradation of sulfur-containing amino acids is catabolized by *Fusobacterium*, *Clostridium*, *Escherichia*, *Salmonella*, *Klebsiella*, *Streptococcus*, *Desulfovibrio*, and *Enterobacter* to produce H₂S as a byproduct [24].

Luminal production of H_2_S can traverse the epithelial barrier and influence distant tumor sites via H_2_S transport mechanisms (e.g. diffusion, carrier-mediated uptake) or via systemic signaling via persulfidation. Although H₂S rapidly diffuses from the colon into the bloodstream, the vast majority is metabolized by colon cells and the liver before it can circulate throughout the body. Colonic epithelium is highly adapted to remove H₂S through mitochondrial sulfide-oxidizing enzymes that convert H₂S into thiosulfate, sulfate, and other metabolites before substantial amounts enter the portal circulation. Additionally, H₂S has a short half-life in blood; systemic effects of H_2_S are more likely mediated through circulating sulfur metabolites (thiosulfate, polysulfides, persulfides) than through direct transport of free H₂S [25,26]. Although moderate increases in luminal H₂S often led to only small increases in circulating free H₂S, very high luminal concentrations may overwhelm local detoxification capacity, thereby increasing systemic exposure.

## 3. Physiological functions of GSH and H_2_S in the mammalian system

To elaborate on the effects, we need to understand how GSH and H_2_S are produced and metabolized in the normal physiological system, and how their metabolism is perturbed in tumors. The main function of GSH and H_2_S is the reduction of oxidative stress directly or indirectly by scavenging free radicals (ROS and RNS). In the next part, these mechanisms of action are described in detail.

### 3.1. Physiological functions of GSH

GSH is found in high concentrations (1–10 mM) across various cellular compartments and is widely recognized as a critical non-enzymatic antioxidant in cellular processes. The enzyme GPX uses GSH as a substrate to produce GSSG by utilizing the thiol (–SH) group of its cysteine residue to interact with ROS or electrophiles (Figure 1B). GSH is also directly involved in glutathione S-transferase (GST) reactions that detoxify xenobiotics [27]. Additionally, GPX uses GSH in reversible glutathionylation/deglutathionylation reactions, thereby protecting proteins from irreversible oxidative damage [9]. To sustain these functions, GSH can be regenerated from GSSG by GR and NADPH, as cofactors (discussed in Section 2.1).

Under oxidizing conditions, the oxidized form of GSH can reversibly bind to protein thiol residues, offering protection against irreversible oxidative damage and potentially modulating protein activity [13]. A study also demonstrated that the glutathionylation of mitochondrial uncoupling proteins UCP2 and UCP3 plays a significant role in regulating mitochondrial ROS levels [28].

GSH is also found to neutralize RNS, such as nitric oxide (NO), a reactive gas produced by the enzyme nitric oxide synthase (NOS). The high levels of GSH in cells facilitate the rapid reaction of NO with the cysteine thiol group of GSH, resulting in the formation of S-nitrosoglutathione (GSNO) [29]. GSNO not only acts as a reservoir for NO bioactivity but also serves as an effective delivery system for NO, playing a crucial role in maintaining cellular homeostasis.

### 3.2. Physiological functions of H_2_S

H_2_S is an intriguing weak diprotic acid that dissociates into hydrosulfide (HS−) and sulfide (S^2−^) anions with pKa1 and pKa2 values of 6.9 and 17, respectively, in aqueous solution [30]. Under physiological pH conditions, such as 7.2 in the cytosol, H_2_S exists in rapid equilibrium with HS−, underscoring its significance in biological processes. This suggests that it can inhibit the harmful effects of ROS and/or RNS on organic substances. The nucleophilic properties of H_2_S enable it to react directly or indirectly with important oxidants, including hydrogen peroxide (H_2_O_2_), peroxynitrite (ONOO^−^), and hypochlorite (CLO^−^), and the superoxide anion (O_2_^−^), all of which are examples of ROS and RNS [31].

**Figure 3.**
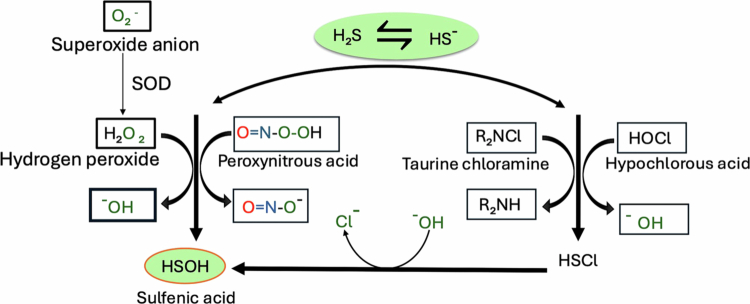
Reactions of H_2_S with oxidants in controlling oxidative stress: H_2_S reacts with oxidants such as hydrogen peroxide, peroxonitrous acid, hypochlorous acid, and chloramines and transforms them into sulfenic acid (an unstable intermediate), which is further converted to polysulfides, elemental sulfur, and sulfate. Abbreviation: HSOH, sulfenic acid.

H_2_S can react with two-electron oxidants such as H_2_O_2_, ONOO^−^, CLO^−^, and chloramines to form sulfenic acid (HSOH), an unstable intermediate [31]. This intermediate then converts to polysulfides, elemental sulfur, and sulfate (Figure 3); the final product specificity depends on the H_2_O_2_-to-H_2_S ratio [31]. These interactions are thermodynamically favorable, indicating that H_2_S plays a crucial role as an antioxidant or ROS/RNS scavenger, thereby contributing to cellular defense against oxidative stress.

Even though these reactions of H_2_S and some oxidants (such as hydroxyl radicals) are very fast, these oxidants can also react with other metabolites (such as sugars, ascorbate, and GSH), which are often present in significantly higher concentrations than H_2_S. Given the numerous studies on the effects of H_2_S in reducing oxidative stress, there also may be indirect mechanisms (as reviewed in [32]) through which H_2_S contributes to cellular health and resilience against oxidative stress.

## 4. Importance of GSH regulation in cancer

### 4.1. Connection of different GSH levels with initiation and progression of cancer

Studies indicate that GSH can play a beneficial role in cancer, depending on the stage of the disease, specifically initiation versus progression stages [33]. In the initiation phase, GSH serves a protective function by preventing DNA oxidation and damage by counteracting ROS [34]. GSH is also found to protect healthy cells by eliminating carcinogens through the action of GSTs [34]. Studies have shown that various cancer cells (including ovarian, breast, and lung cancers) commonly exhibit elevated intracellular GSH levels via various mechanisms to balance elevated ROS levels [35]. This upregulation of GSH levels in specific cancer types helps safeguard cancer cells from oxidative stress-induced apoptosis and ferroptosis.

### 4.2. Importance of GSH regulation in cancer progression

Research indicates that limiting cysteine through cyst(e)inase (a modified enzyme that degrades cysteine and cystine) or by altering SLC7A11 expression (a cystine/glutamate transporter protein) significantly enhances ferroptosis in pancreatic cancer cells [36,37]. This process results from the depletion of intracellular GSH and the subsequent elevation in ROS levels [36,37]. In renal cell carcinoma, notable alterations in GSH metabolism were observed, characterized by increased expression and activity of the GCLC and GCLM enzymes [38]. This elevation in GSH levels can be explained by the loss of VHL (von Hippel-Lindau) function, which is common in many renal cancers and stabilizes HIF-α (hypoxia-inducible factor), thereby creating a ‘pseudohypoxic’ environment that increases the activity of GCLC and GCLM [39]. Furthermore, squamous cell carcinoma exhibits a marked increase in the expression of numerous enzymes involved in the GSH metabolic pathway, underscoring the crucial antioxidant function of GSH in this cancer type [40]. Cancer cells found to have increased PPP flux, producing increased levels of NADPH, which then serves as a cofactor for GSH production [38]. Indeed, a study showed that clear cell renal cell carcinoma (ccRCC) cells have significantly higher accumulation of GSH and PPP-related metabolites [38]. The adaptations associated with high GSH content in cancer cells are thought to be essential for promoting tumor growth and overcoming drug resistance. A comprehensive analysis of 928 distinct cancer cell lines from 20 major cancer types reveals that the GSH/GSSG ratio and NADP^+^ levels are closely linked to constitutive activation of a regulator of oxidative stress response transcription factor Nuclear factor erythroid 2-related factor-2 (NRF2) via Kelch-like ECH-associated protein-1 (KEAP1) mutations [41].

### 4.3. Importance of GSH transport between organelles in cancer cells

Given its small size and net negative charge, GSH transport typically requires specialized carriers to cross the plasma membrane and organelle membranes to reach its various targets. Furthermore, because GSH is the most abundant non-protein thiol and a key cellular metabolite, the need for effective GSH transport is even more pronounced in cancer cells. Studies have shown that MRP1 (multidrug resistance-associated protein 1) plays a key role in conferring multidrug resistance (MDR) in tumor cells by exporting xenobiotics, a function that is intricately linked to GSH [27]. Various organelles, including the nucleus, endoplasmic reticulum (ER), and mitochondria, require their own specialized GSH pools [8]. Based on the redox status (GSH/GSSG ratio) of an organelle, GSH can be transported between the organelles. For instance, mitochondria contain approximately 10%–15% of the total cellular GSH content, which is particularly important because they generate high levels of ROS during oxidative phosphorylation (OXPHOS) [42]. Within the nucleus, GSH significantly contributes to cancer cell proliferation by neutralizing free radicals and alternatively reducing DNA damage [43]. Notably, the nuclear GSH pool appears to be dynamic, exhibiting significant fluctuations in concentration throughout the cell cycle. Hence, effective GSH transport is crucial for cancer cells, given their higher proliferation rates and high energy demands.

### 4.4. Importance of GSH regulation in the TME

Beyond its role in cancer cells, GSH also modulates the TME, potentially favoring cancer cell survival and growth. For example, studies have found that GSH can promote lipid accumulation and storage and support a TME that favors tumor growth in obesity-induced malignancies [44,45]. These studies found that lowering GSH levels, either genetically or pharmacologically, prevents obesity induced cellular transformation [44,45].

Hypoxia (a key feature of the TME) results from impaired angiogenesis and is associated with metabolic pathway changes, notably increased dependence on glycolysis, which leads to tissue acidosis [46]. Redox reactions depend on pH, with the redox potential rising as pH decreases [47]. Extracellular acidosis in the TME, commonly observed in solid tumors [48], may intensify oxidative stress and related damage. Indeed, previous studies have documented heightened lipid peroxidation during tissue acidosis [49]. While strong antioxidant defenses usually maintain oxidative stress in cancer cells, healthy cells not adapted to such oxidative conditions can be severely affected, potentially leading to toxicity and mutations. This indicates that an acidic TME may promote tumor growth by damaging the normal tissues that are being invaded.

Acidic TME has been associated with increased exosome release and entry into the metastatic stage [50]. A study showed that GSH levels were higher in glioblastoma (GBM) cell lines than in their respective exosomes, whereas they found GSSG was concentrated in exosomes [51]. Research on various human tumor cell lines, including those from colon, breast, melanoma, osteosarcoma, and prostate cancers, indicates that pH changes from 7.4 to 6.5 correlate with increased exosome release [52]; hence, this suggests that cancer cells may release excess GSSG in the TME to maintain high GSH/GSSG ratios.

Cancer stem cells (CSCs) are a small subpopulation of cells within tumors that drive tumor growth by promoting a cancer-favorable TME. A study found that oxidative stress-mediated NRF2 activation promoted GSH production in CSCs, thereby inducing differentiation of CSCs into bulk tumor cells [53]. The pro-tumor function of T cells in the TME creates a significant barrier to effective antitumor responses. A study showed that Tregs (immune-suppressive T cells) inhibit cysteine release from dendritic cells (DCs) via a CTLA-4-dependent mechanism [54]. This reduction in cysteine levels lowers GSH levels in T cells, thereby negatively affecting T cell activation.

### 4.5. Significance of GSH in therapeutic resistance in cancer treatment

Chemoresistance and radiotherapy resistance remain major barriers to effective cancer treatment. Because many therapeutic regimens heavily rely on oxidative damage for their antitumor effects, high intratumoral GSH content is frequently correlated with treatment failure and adverse prognosis [55]. Tumor cells found to buffer therapy-induced oxidative stress and attenuate drug-induced cytotoxicity when GSH levels increase, and GSH-dependent enzymes are activated [56]. This redox-adaptive phenotype was found to be associated with cisplatin resistance. Elevated intracellular GSH levels, often driven by BCL-2 overexpression or NRF2 activation, promote resistance primarily by suppressing apoptotic commitment rather than by preventing cisplatin-induced DNA damage [43,57].

As GSH plays a crucial role in chemotherapy resistance, its inhibition in combination therapies has been shown to enhance the efficacy of chemotherapies [58]. Similarly, chronic exposure to ROS-generating agents, such as etoposide (a chemotherapeutic drug to which many patients develop chemoresistance), has been shown to induce chemoresistance in a neuroblastoma cell line (HTLA-Chr) by increasing intracellular GSH, whereas pharmacological inhibition of cystine uptake via cystine–glutamate antiporter transport system (xCT) reduces GSH synthesis and restores drug sensitivity [59,60]. More broadly, chemotherapy-induced oxidative stress favors the selection of GSH-high tumor subpopulations, a process that can be therapeutically exploited by targeting GSH biosynthesis [61].

Clinically, excessive GSH intake has been associated with higher recurrence rates and chemoresistance in breast cancer patients, with resistant tumors exhibiting elevated antioxidant capacity [62]. Recent studies further identify the NRF2–SCL7A11 axis and downstream GPX4 activity as central mediators of GSH-driven chemoresistance in multiple tumor types, including esophageal and colorectal carcinomas [63,64], highlighting this pathway as a therapeutic vulnerability for sensitizing tumors to specific targeted therapies and chemotherapeutics [63,64]. On the other hand, due to the high concentration of GSH in many tumors and its high reactivity, GSH is used as an activator of prodrugs (e.g. romidepsin), which are used to treat cutaneous T-cell lymphoma and other peripheral T-cell lymphomas [58].

## 5. Importance of H_2_S regulation in cancer

### 5.1. Connection of different H_2_S levels with initiation and progression of cancer

H_2_S can be both beneficial and harmful to health, exhibiting a bell-shaped dose-response curve [65]. At low concentrations (mainly produced endogenously), H_2_S plays a protective role by reducing oxidative stress and inflammation and inducing neoangiogenesis, whereas at higher concentrations, it can have detrimental effects on health [5,6]. Endogenous production or low exogenous production of H_2_S promotes cancer cell growth by stimulating angiogenesis, increasing mitochondrial bioenergetics, and enhancing antioxidant activity [66–69]. While H_2_S at higher concentrations prevails over its cytoprotective effects, H_2_S concentrations above a certain threshold exert anticancer effects by inducing apoptosis and DNA damage and inhibiting cell cycle progression [70–72].

### 5.2. Importance of H_2_S regulation in cancer progression

H_2_S is increasingly recognized for its involvement in both the development and progression of various types of cancer. In colon cancer, a study showed that tumor-derived H_2_S (produced mainly via the transsulfuration pathway) plays a pivotal role by stimulating bioenergetics, promoting cell proliferation, and facilitating peritumoral angiogenesis [66]. This study suggests that in colon cancer, CBS is overexpressed, producing H_2_S at low to moderate levels in the early stages of cancer, and acts as a bioenergetic factor that supports cancer cell growth and promotes vasorelaxation, thereby contributing to tumor growth and proliferation. Bhattacharyya and colleagues found that CBS co-localizes with mitochondrial markers in ovarian cancer, emphasizing the importance of this enzyme in cancer cell metabolism [67]. This study suggests that reducing H_2_S production via CBS inhibition diminishes cellular GSH levels, activates the tumor suppressor p53, and inhibits NF-κB, collectively inhibiting ovarian tumor growth and reducing cisplatin resistance.

Similarly, several studies have investigated the mechanisms by which H_2_S promotes cancer growth by treating various cancer cell types with H_2_S donor compounds (e.g. NaHS, GYY4137). For instance, in oral squamous cell carcinoma (SCC), low concentrations of NaHS (a H_2_S donor compound) have been associated with increased levels of p-Akt and p-ERK1/2, which promote cell proliferation by facilitating SCC cell entry into S phase [68]. Similarly, a study demonstrates that the p38 MAPK/ERK1/2-COX-2 pathway plays a pivotal role in NaHS-induced proliferation and anti-apoptotic effects in C6 glioma cells [69]. Additionally, in the hepatocellular carcinoma (HCC) cell line PLC/PRF/5, the exogenous application of 500 μM NaHS inhibits caspase-3 production and activates the NF-κB pathway, promoting cell proliferation and inhibiting apoptosis via the STAT3/COX-2 signaling pathway [73]. Furthermore, in esophageal cancer cell line EC109, 400 μM NaHS significantly reduces apoptosis by activating NF-κB and the p38 MAPK/ERK1/2-COX-2 signaling pathways [74]. Moreover, treating multiple myeloma cells with 500 μM NaHS for 24 h promotes cell proliferation and migration by upregulating p-Akt expression [75]. Concentrations of 50–200 μM NaHS have been shown to induce proliferation of human colon cancer cells by promoting phosphorylation of Akt and ERK, while inhibiting p21 expression and nitric oxide production [76].

Three main transsulfuration enzymes (CBS, CTH, and 3MST) are involved in endogenous H_2_S production, and they are differentially expressed and active in a tissue-specific manner in the mammalian system. Interestingly, many studies have reported a positive association between the expression of CBS, CSE, and 3MST with the progression of various cancer types, as summarized in Table 1.

**Table 1.** Study the association of H_2_S-producing enzymes CBS, CSE, and 3MST with tumor progression across human cancers.

Enzymes	Cancer type	Clinical/pathological findings	References
CBS	Ovarian cancer	Elevated CBS levels were linked with high-grade ovarian tumors	[67]
CBS, CSE	Breast cancer	Higher CBS and CSE expression compared with adjacent healthy tissue; correlated with disease progression	[77,78]
CBS, CSE	Gastric carcinoma	CBS and CSE expression significantly increased in gastric carcinoma	[79]
CBS, CSE, 3MST	Bladder urothelial carcinoma	Increased CBS, CSE, and 3MST expression; High CBS and 3MST expressions are associated with the advanced stage	[18,80]
CBS, CSE, 3MST	Lung adenocarcinoma/NSCLC	Higher levels of CBS, CSE, and 3MST were observed compared with adjacent lung tissue	[81,82]
CBS	Renal tumors	High CBS in renal oncocytoma and urothelial carcinoma compared with benign samples	[83]
CBS, CSE	Colorectal cancer	CBS upregulated in colorectal adenoma & carcinoma; whereas CSE was unchanged	[84]
CBS, CSE, 3MST	Papillary thyroid carcinoma	CBS, CSE and 3MST expression were studied, only CSE is elevated, associated with tumor size and metastasis	[85]
CBS	Thyroid carcinoma	CBS is strongly expressed in carcinomas but not in benign tissue	[86]
CBS, CSE	Multiple myeloma	CBS elevated in the patient's bone marrow; CSE unchanged	[87]
CBS, CSE, 3MST	Renal cell carcinoma	All three enzymes were upregulated compared with healthy tissue	[88]
CSE	Acute lymphoblastic leukemia	Higher CSE expression in PBMCs of patients	[89]
CBS	Hepatocellular carcinoma	CBS decreased in tumors; low CBS predicts worse survival	[90]
CBS	Glioma	Low CBS correlates with poor outcomes	[90]

Abbreviations: CBS, cystathionine β synthase; CSE, cystathionine-γ-lyase; 3MST, 3-mercaptopyruvate sulfur transferase.

### 5.3. Importance of H_2_S regulation in the TME

Endogenously and exogenously produced H_2_S has recently been identified as a vital signaling molecule within the TME, influencing angiogenesis, immune regulation, and stromal remodeling [91]. As a proangiogenic factor, H_2_S has been found to increase neo-vascularization in tumors, which contributes to the progression and resistance in several cancer models, including ovarian [67], prostate [92], and breast [77] cancers. In a study, Sen et al. showed that CBS overexpression protects breast cancer cells from activated macrophages; however, normal CBS expression did not confer this benefit [77]. Additionally, proangiogenic effects of H_2_S via PI3K/AKT, MAPK, and KATP pathways suggest that H_2_S improves the TME, thereby favoring tumor cells by increasing nutrient and oxygen availability [91].

M1 macrophages are well known for their antitumor roles, while M2 macrophages contribute to pro-tumorigenic outcomes. As H_2_S has been found to promote macrophage transition from M1 to M2 [93], endogenous H_2_S production may protect tumor cells via M1-to-M2 polarization.

Regulatory T (Treg) cells are a specialized subset of T cells characterized by expression of the FOXP3 (a transcription factor) and by their ability to suppress immune responses. Research indicates that H_2_S is essential for the differentiation of Treg cells, achieved through persulfidation of LKB1 (liver kinase B1) and activation of AMPK-1 (adenosine monophosphate-activated protein kinase 1) [94,95]. Additionally, H_2_S influences Treg cells by modulating chromatin remodeling [96]. For instance, persulfidation of the nuclear transcription factor Y subunit beta (NFYβ) enhances its binding to the promoters of TET1 and TET2 (ten-eleven translocation 1/2), thereby increasing their expression, which mediates FOXP3 demethylation to drive differentiation of Treg cells [96]. This process results in FOXP3 demethylation, promoting Treg differentiation and reducing autoimmunity. Importantly, mice lacking CBS exhibit fewer regulatory T cells and marked inflammatory infiltration, effects that can be reversed by H_2_S donor supplementation [96].

### 5.4. Significance of H_2_S in therapeutic resistance in cancer treatment

Studies have found that low, physiologically relevant levels of H₂S promote tumor cell survival and attenuate the cytotoxic effects of chemotherapy and radiotherapy [66,97]. H_2_S has been implicated in the development of acquired resistance to chemotherapeutic agents (e.g. 5-fluorouracil) and immunotherapies (anti–PD-L1 and anti–CTLA4) [98,99]. Because apoptosis can be reduced by persulfidation, which activates key pro-survival signaling pathways, including NF-κB, PI3K-AKT, and NRF2, H_2_S is believed to mediate resistance primarily through protein persulfidation [17]. In addition, low levels of H_2_S support mitochondrial bioenergetics and reduce oxidative stress, thereby also creating an anti-apoptotic effect on cancer cells [66].

Importantly, H₂S signaling intersects with DNA damage response pathways and promotes resistance to genotoxic therapies [100]. Moreover, elevated H₂S enhances DNA repair efficiency and protects against oxidative DNA damage, thereby reducing sensitivity to chemotherapeutic agents, including platinum compounds and topoisomerase inhibitors [100]. Clinical samples and preclinical models have shown that increased expression of H₂S-producing enzymes is associated with poor prognosis, tumor aggressiveness, and diminished therapeutic response in colorectal, breast, lung, ovarian, and other cancers [67,101]. Together, these findings position H₂S metabolism as a central redox-adaptive mechanism driving therapeutic resistance, and identifying H₂S biosynthesis inhibitors could be a potential strategy to overcome treatment failure.

Beyond the direct effects of GSH and H₂S in the genesis and progression of different types of cancer, as discussed above, these two metabolites indirectly protect cancer cells from lipid peroxidation and ferroptosis. In this aspect, GSH and H₂S are found to coordinate an adaptive biochemical shield that safeguards aggressive tumors against iron-dependent and lipid peroxidation-driven cell death. In the following section, we discuss how GSH and H_2_S regulate this same function through distinct molecular mechanisms and how targeting these mechanisms is a promising therapeutic strategy for killing cancer cells.

## 6. Importance of GSH and H_2_S in suppression of lipid peroxidation and ferroptosis in cancer cells

**Figure 4.**
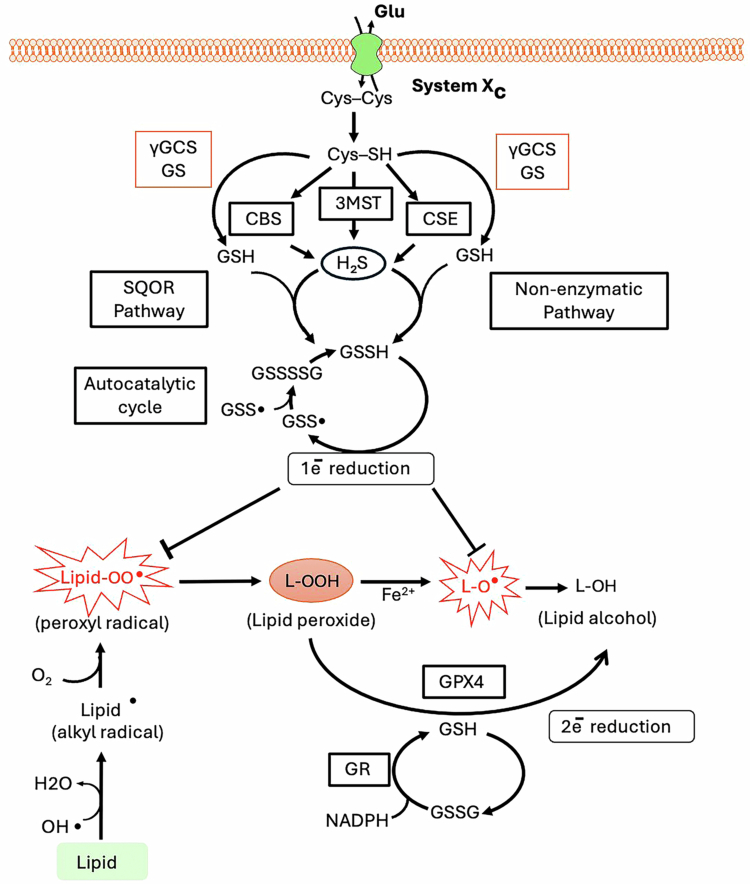
Schematic illustration of lipid peroxidation and GSH and H_2_S mediated scavenging mechanism: Reactive oxygen species (ROS) take electrons from membrane lipids, producing water and an alkyl radical. This alkyl radical readily reacts with oxygen to form a peroxyl radical, which is then converted into a lipid peroxide (L-OOH). The GSH/GPX system detoxifies lipid peroxides and creates lipid alcohols through a 2-electron reduction, while GSH-persulfide (GSSH) can inhibit lipid peroxidation via a 1-electron reduction. Conversely, Fe²⁺ promotes the buildup of lipid peroxides through the Fenton reaction. Abbreviations: γGCS, γ-glutamylcysteine synthetase; GS, glutathione synthase; GR, glutathione reductase; GSH, glutathione; GSSH, glutathione persulfide; GSS⋅, perthiyl radical, GSSSSG, glutathione tetrasulfide; CBS, Cystathionine β Synthase; CSE, cystathionine-γ-lyase; 3MST, 3-mercaptopyruvate sulfur transferase; SQOR sulfide quinone oxidoreductase; GPX, glutathione peroxidase.

During lipid peroxidation, different species of ROS take electrons from polyunsaturated fatty acids of the lipid membrane and produce water and an alkyl radical. This fatty acid radical reacts readily with oxygen to generate a peroxyl radical, which is further converted into lipid peroxide via transfer of a hydrogen atom from the organic substrate. Studies have shown that sulfur from cysteine is released as H_2_S, which then reacts with GSH to form a GSH persulfide (GSSH) [102]. The unique structure of GSSH allows it to donate a single electron to lipid radicals via hydrogen-atom transfer, thereby preventing the propagation of lipid peroxidation (Figure 4). The formed perthiyl radical (GSS⋅) reacts with another perthiyl radical, producing GSH tetrasulfide (GSSSSG), which can then be reduced by GR or react directly with GSH to restore GSH persulfide levels. In addition, GSH has been found to serve as a cofactor for GPX4, facilitating the reduction of lipid peroxides via a 2-electron reaction [103]. Because endogenous GSH and GSSH production is the primary means of suppressing lipid peroxidation, many studies have focused on inhibiting endogenous GSH and H_2_S production as a potential anticancer strategy.

Ferroptosis is caused by excessive lipid peroxidation in the membrane. Although in normal cells, it is controlled by normal cellular antioxidant levels, in cancer cells, due to their elevated iron demands and increased ROS production during rapid growth, they exhibit heightened susceptibility to ferroptosis [104,105]. Interestingly, in many cases, cancer cells are found to suppress ferroptosis through various mechanisms to survive and promote growth. Transsulfuration is closely linked to ferroptosis [106], and studies have shown that cancer cells upregulate various transsulfuration enzymes to counteract oxidative stress-induced ferroptosis [107]. Indeed, recent studies have found that cancer cells upregulate GSH and H_2_S production, thereby inhibiting ferroptosis by scavenging excessive ROS [108,109]. GPX4 has been shown to use GSH to reduce organic hydroperoxides and lipid peroxides in cell membranes to harmless alcohols, thereby preventing cell damage via ferroptosis (Figure 4) [110]. GSH additionally supports the regeneration of alpha-tocopherol, an essential factor in preventing lipid peroxidation within cell membranes [111].

H_2_S has been shown to exert protective effects against ferroptosis by chelating redox-active ferrous iron, regulating iron-handling proteins, restoring GSH levels, and suppressing ROS generation. A study showed that H_2_S inhibits NOX2 activity, thereby protecting retinal ganglion cells from pressure-induced ferroptosis [102]. The key role of H_2_S in ferroptosis suppression is attributed to its activation of iron homeostasis, mitochondrial function, and ROS-scavenging pathways [112]. Additionally, H_2_S regulates iron metabolism by modulating transferrin receptor 1 (TfR1) and ferroportin (Fpn1) via the IL-6/STAT3/hepcidin axis, thereby preventing excessive iron accumulation and ROS-induced ferroptosis under inflammatory conditions [113]. It is important to note that H_2_S can modulate ferroptosis in cancer in a context-dependent manner, depending on H_2_S levels. In colorectal cancer, endogenous H_2_S production is elevated due to cystathionine β-synthase (CBS) overexpression. Conversely, exogenous H_2_S stabilizes the cystine transporter xCT through persulfidation of OTUB1 at cysteine 91, thereby inhibiting ferroptosis in colon cancer cells [114]. Notably, a study found that scavenging H_2_S using virus-like silica zinc oxide (VZnO) nanoparticles promotes ferroptosis and suppresses tumor growth, highlighting the pro-tumorigenic role of H_2_S in this context [115].

## 7. Importance of diet in the regulation of GSH and H₂S levels in cancer

Among established dietary patterns, the Mediterranean diet has been consistently linked with a lower overall burden of cancer and with improved clinical outcomes across multiple malignancies, including obesity-associated and organ-specific tumors [116,117]. This dietary profile prioritizes whole grains, legumes, nuts, a wide spectrum of fruits and vegetables, regular intake of fish and seafood, restrained consumption of lean white meats and dairy products, and olive oil as the principal lipid source. Collectively, these components ensure a high dietary intake of fiber, polyphenols, and unsaturated fatty acids, which converge on anti-inflammatory signaling and the reinforcement of endogenous antioxidant defenses [116,117]. In contrast, Western dietary patterns—characterized by excess red and processed meat, refined carbohydrates, and saturated fats—are robustly associated with heightened risk of colorectal and several other cancers [5]. Mechanistically, this association is partly attributable to increased exposure to heme iron, N-nitroso compounds, and pro-oxidant lipid species, which intensify oxidative stress and mutagenic pressure [118,119].

At a molecular level, the methionine cycle serves as a central metabolic nexus linking dietary inputs to redox regulation and malignant transformation. Dietary methionine is converted to S-adenosylmethionine (SAM), the universal methyl group donor required for DNA, RNA, and histone methylation, thereby exerting profound control over the epigenetic programming of both tumor and immune cells [120,121]. Rapidly dividing cancer cells exhibit an exaggerated dependence on one-carbon metabolism to sustain nucleotide biosynthesis, maintain NADPH production, and support antioxidant systems (e.g. GSH, H_2_S), often resulting in a state of methionine addiction [122].

### 7.1. Importance of methionine and cysteine restriction in cancer

Cysteine is the primary precursor of GSH and H_2_S biosynthesis; the availability of cysteine can be a rate-limiting factor for the production of these non-enzymatic antioxidants. Based on the above discussion, it is clear that cysteine bioavailability can affect the metabolic fitness of cancer cells and may influence the development of therapy resistance. Although cysteine can be derived from protein breakdown or from methionine via the methionine cycle, most cellular cysteine is obtained from the diet as the oxidized form. It is not surprising that increased expression of many cysteine transporters (e.g. xCT, SLC3A1) is associated with higher intracellular GSH levels, poor prognosis, and drug resistance. It is also reported that expression of the main cysteine transporter xCT is regulated by NRF2, a master regulator of the cellular redox state [123]. Although blocking these cysteine transporters (xCT) appears to be a viable target for cancer therapy, cysteine can also be produced from methionine via the methionine cycle. Through the transsulfuration pathway, homocysteine is irreversibly converted to cystathionine and subsequently to cysteine, thereby directly coupling methionine availability to cysteine-dependent biosynthesis of both GSH and H₂S [124]. Restriction of methionine via dietary modulation, therefore, simultaneously attenuates cellular methylation capacity while limiting the sulfur amino acid flux that sustains antioxidant buffering and gasotransmitter signaling. This dual constraint exposes a metabolic vulnerability in tumors that are highly dependent on these interconnected networks [125,126].

Consistent with this framework, dietary methionine restriction has emerged as a compelling strategy to sensitize tumors to oxidative injury and ferroptotic cell death. In preclinical models, intermittent methionine deprivation markedly enhances intratumoral ferroptosis and potentiates immune checkpoint blockade by constraining cysteine and GSH availability while amplifying lipid peroxidation [127]. Parallel systems-level analyses and early translational studies further suggest that methionine restriction remodels tumor metabolic architecture and reprograms T-cell function, potentially improving responsiveness to immunotherapy [125,128].

Plant-exclusive dietary patterns, including vegan diets, which are typically lower in total protein and sulfur-containing amino acids, have been associated with reduced circulating GSH concentrations relative to omnivorous or lacto-ovo-vegetarian diets, suggesting that low-methionine dietary regimens may be systematically exploited to modulate redox tone under selected clinical conditions. However, large-scale population studies remain heavily reliant on self-reported dietary intake and food-frequency questionnaires, which introduce substantial measurement error and complicate causal inference regarding specific nutrient–cancer relationships [129].

The gut microbiome (in a commensal relationship) breaks down indigestible dietary fiber and other compounds into vital substances that influence host metabolism, immune function, and overall health. Diet profoundly affects the gut microbiome; studies have shown various dietary patterns correlating with distinct gut microbiota compositions [130–132]. Increased protein intake enhances colonic delivery of undigested nitrogen and sulfur, thereby expanding the substrate pool available to sulfidogenic bacteria and increasing H₂S production, even in the absence of major shifts in microbial composition [133]. It is important to maintain a balance in H_2_S production via microbiota, as excessive levels can compromise mucosal integrity. This process is thought to occur through the breakdown of mucosal disulfide bonds [134], the inhibition of butyrate oxidation in colonocytes by cytochrome c [135], and potential genotoxic effects [136]. Notably, the association of *Fusobacterium nucleatum*, a recognized H_2_S-producing bacterium in the gut, with colonic tumors underscores the gut microbiome's role in shaping gut health via H_2_S production [21].

## 8. Metabolic reprogramming linking GSH and H_2_S production in cancer

Metabolic reprogramming (a hallmark of cancer) is a fundamental alteration in the energy metabolism of cancer cells that enables their rapid growth, proliferation, and survival. Here, we discuss various instances in which cancer cells rely heavily on the transsulfuration pathway for GSH and H_2_S production via metabolic reprogramming.

Cancer cells rely heavily on one-carbon metabolism to produce metabolites required for tumor growth; this reliance is even more pronounced in response to metabolic challenges imposed by genetic mutations. For instance, K-ras G12V mutations cause mitochondrial dysfunction, leading to decreased oxidative phosphorylation and increased ROS production [137]. In response, cancer cells shift from oxidative phosphorylation to glycolysis to meet their energy needs, and, in parallel, the activity of one-carbon metabolism is enhanced to cope with oxidative stress by producing GSH and H_2_S. This integrated metabolic reprogramming enables tumor cells to maintain growth and survival, even in nutrient-poor environments, by balancing energy production with the demand for biosynthetic intermediates such as nucleotides and amino acids [137,138].

To maintain a high proliferation rate, growth, and consistent energy production, many cancer cells are often found to be addicted to methionine [4]. In addition to producing essential metabolites, the methionine cycle generates the toxic amino acid homocysteine, which is further implicated in cellular pathology through oxidative stress, homocysteinylation of proteins, and lipid peroxidation [6]. Interestingly, cancer cells shuttle excess homocysteine to the transsulfuration pathway, which produces GSH and H_2_S to counteract excess homocysteine-mediated toxicity [139]. Indeed, many studies have found that cancer cells overexpress CBS (8–10), the first (and rate-limiting) enzyme in the transsulfuration pathway [140], which produces the cellular primary antioxidants GSH and H_2_S. Through this mechanism, cancer cells not only tolerate oxidative stress but also survive and grow under cellular stress conditions.

Hypoxia is a hallmark of tumors and can lead to a temporally increased production of ROS. Cancer cells have been found to activate specific transcription factors (FOXO, Nrf2, HIF-1, NF-κB, and p53) to overcome oxidative stress induced by hypoxia [141–143]. Many of these transcription factors regulate the synthesis of GSH and H_2_S [141–143]. Additionally, cancer cells have been found to create a ‘pseudohypoxic’ environment that activates HIF-1, which in turn induces GSH production by regulating the activity of GCLC and GCLM [39].

Interestingly, in cancer cells, metabolic reprogramming during oxidative stress can be independent of the inducers and be modulated by multiple factors. For example, hypoxia-induced oxidative stress may activate modulators HIFs, NRF2, and FOXO [141–143], whereas p53 deletion-induced oxidative stress may activate modulators NRF2 and NF-κB [144,145]. Additionally, under oxidative stress conditions, cancer cells activate GCL via NRF2 (a regulator of antioxidant pathways) and HIF1 (a sensor of hypoxia) signaling pathways to produce GSH. In breast cancer, NRF2 was found to be a key regulator of chemotherapeutic resistance under hypoxia via the ROS-Nrf2-GCLC-GSH pathway [146].

The ‘Warburg effect’ is a hallmark of cancer, in which cancer cells reprogram their metabolism to favor glycolysis over mitochondrial oxidative phosphorylation, even when oxygen and functional mitochondria are available. H_2_S has long been known to inhibit the mitochondrial respiratory chain by affecting cytochrome c oxidase. Some studies suggest that at low doses, H_2_S can act as a bioenergetic stimulator of the respiratory chain [147]. It has been reported that endogenous H_2_S enhances glucose uptake, modulates LDH-A activity through persulfidation, and increases ATP production, thereby promoting both glycolysis and oxidative phosphorylation pathways [147].

**Figure 5.**
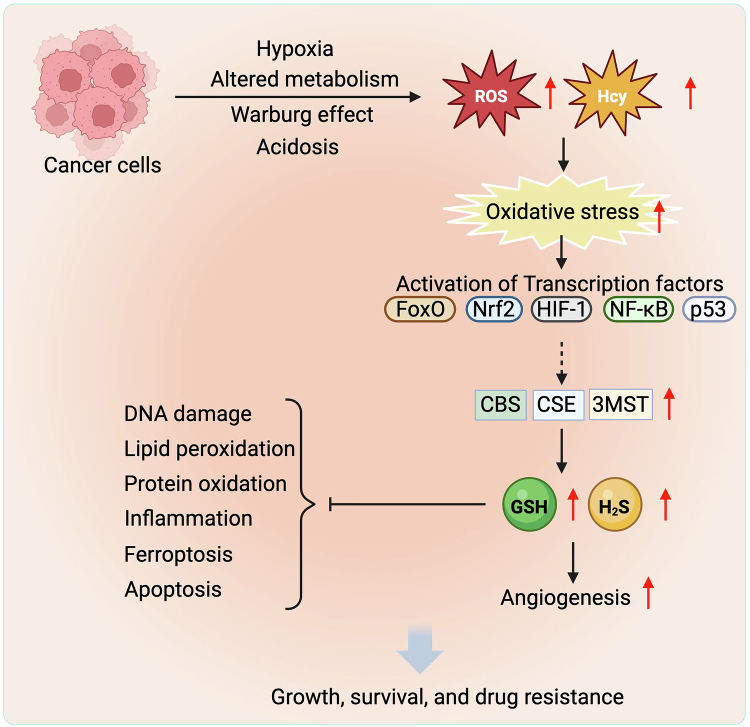
Metabolic reprogramming for GSH and H_2_S production in cancer cells. Tumor cells produce high levels of ROS and homocysteine (Hcy), thereby activating oxidative stress responses mediated by various transcription factors. These activations are linked to increased production of GSH and H_2_S via altered expression of CBS, CSE, and 3MST, which promotes the growth and survival of cancer cells. Abbreviations: ROS, reactive oxygen species; Hcy, homocysteine; GSH, glutathione; CBS, cystathionine β synthase; CSE, cystathionine-γ-lyase; 3MST, 3-mercaptopyruvate sulfur transferase. The Figure was created using BioRender.

As discussed above, specific cancer cells have been found to reprogram their metabolism to produce GSH and H_2_S to support rapid energy production and cell proliferation, and to overcome oxidative and extracellular stress (such as low nutrient availability) and therapeutic challenges (Figure 5).

## 9. Summary and conclusions

Collectively, dietary patterns that integrate moderate protein intake with abundant dietary fiber and organosulfur-rich plant foods appear most effective in maintaining a favorable balance between host-protective and tumor-promoting GSH and H₂S pathways. From a cancer-preventive perspective, minimally processed, Mediterranean-like, plant-forward diets are likely to suppress sulfidogenic microbial activity while reinforcing systemic redox stability. In established malignancies, however, more precise dietary modulation of methionine, cysteine, and microbial sulfur metabolism may be required to strategically exploit redox vulnerabilities and enhance therapeutic efficacy.

**Figure 6.**
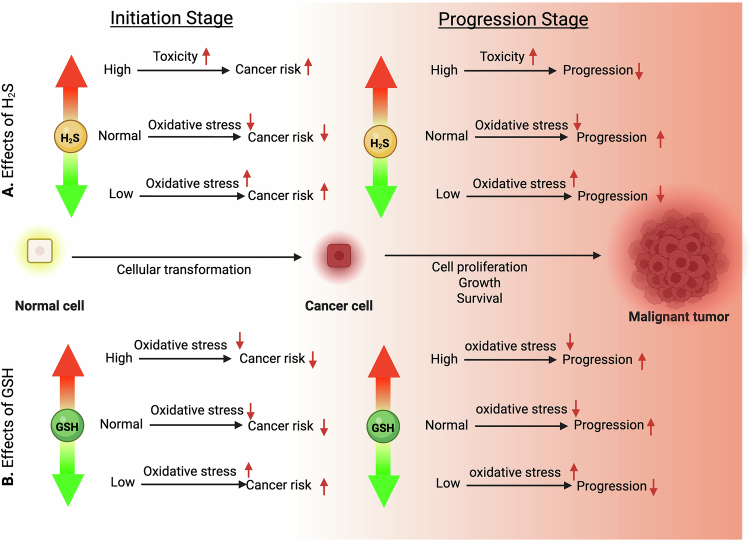
A two-panel diagram shows the effects of H2S and GSH on cancer initiation and progression at high, normal, and low levels. Dual concentration-dependent roles of H_2_S and GSH in tumor initiation vs progression. (a) The top panel illustration shows that physiologically relevant levels of H₂S protect cells by reducing oxidative stress; thus, normal levels of H₂S reduce cancer risk, whereas low levels of H₂S may increase it. However, in tumor cells, normal physiological levels of H_2_S promote survival and progression by reducing oxidative stress, whereas low levels may have the opposite effect. High levels of H_2_S are toxic to tumor cells, as demonstrated by H_2_S donor intervention. (b) Similarly, the bottom panel illustration shows that physiological levels of GSH contribute to cellular protection by limiting oxidative damage and reducing cancer risk; therefore, low GSH levels are associated with increased cancer risk. In contrast, elevated GSH levels promote tumor progression, whereas low levels have the opposite effect in tumor cells. Abbreviations: GSH, glutathione; H_2_S, hydrogen sulfide. The Figure was created using BioRender.

In addition to diet, multiple other factors (e.g. lifestyle, genetics, and disease states) can contribute to oxidative stress, which, in turn, can drive cellular transformation via various mechanisms (e.g. DNA damage, oncogene upregulation, and metabolic abnormalities) [4,5]. GSH represents the dominant intracellular thiol buffer and a central determinant of redox equilibrium, xenobiotic detoxification, and cellular survival. During the premalignant phase, sufficient GSH availability protects against oncogenic transformation by neutralizing electrophiles, restricting ROS-driven DNA damage, and supporting phase II conjugation reactions. Following tumor initiation, cancer cells require more GSH than normal cells, and elevated GSH pools promote tumor cell proliferation, reinforce therapeutic resistance, and blunt ROS-mediated cytotoxicity (Figure 6) [35,148].

H_2_S is now firmly recognized as a gaseous signaling molecule governing vascular regulation, mitochondrial energetics, inflammatory signaling, and cell survival. In oncogenesis, H₂S exhibits a biphasic, concentration-dependent profile: low-to-moderate endogenous levels typically support tumor growth and angiogenesis, whereas higher concentrations exert cytotoxic and pro-apoptotic effects (Figure 6) [66,149,150]. Dietary influences on H₂S homeostasis operate through two principal mechanisms: modulation of endogenous synthesis from cysteine via host enzymatic pathways, and regulation of microbial H₂S production from sulfur-containing substrates within the intestinal lumen [151].

Metabolic heterogeneity, driven by intrinsic and extrinsic factors, contributes to the distinct metabolic characteristics and vulnerabilities of different tumor subtypes [152]. This diversity in metabolic phenotypes is observed even among tumor cells that share similar genetic backgrounds or tissue origins [41]. As cancer cells rely on the methionine cycle for cellular turnover, which produces the toxic amino acid homocysteine, excessive cellular homocysteine production is further implicated in inducing cellular pathology, mainly through oxidative stress, protein oxidation, inflammation, lipid peroxidation, and ferroptosis [6]. Indeed, studies found that cancer patients show high homocysteine levels, but that does not mean that high homocysteine is a risk factor for cancer; instead, cancer cells shuttle excess homocysteine to the transsulfuration pathway (which bifurcates from the methionine cycle) for the production of the most important cellular antioxidants, GSH and H_2_S [139]. Although various drugs (including methotrexate, pemetrexed, palyatrexate, raltitrexed, and 5-FU) that disrupt one-carbon metabolism have long been used in cancer treatment, they have been associated with substantial side effects due to their effects on normal healthy cells [153].

## 10. Limitations

In this manuscript, we present numerous preclinical studies on methionine deprivation or restriction across different cancer cell types; however, the clinical efficacy of dietary methionine restriction remains poorly studied. Although H_2_S is not well studied in various cancers as an antioxidant agent, like GSH, it shares a common metabolic pathway for synthesis and function. Additionally, the role of H_2_S in cancer and its concentration range in human blood are controversial due to measurement limitations; hence, based on the current literature, we sought to demonstrate its relevance to cancer risk and progression. Moreover, given the scope of this review, we did not elaborate on how different diets regulate GSH and H₂S, or on how they modulate cancer risk or progression. However, we tried to shed some light on how diet is also linked to this scenario. Besides, sulfur species have essential roles in cellular redox homeostasis, particularly through the dynamic formation and reduction of protein per/polysulfides, which impact enzymatic functions. However, given the broader focus of this review, we put less emphasis on protein persulfidation in relation to H_2_S metabolism in cancer.

As cancer continues to be a major health concern, one area of increasing interest is a better understanding of the unique mechanisms of cancer cell metabolism that may be exploitable. Based on the above discussion, it is clear that vulnerability to oxidative stress could be a promising anticancer strategy; however, it has adverse effects, as normal cells are not adapted to this oxidative stress environment. Hence, we need a deeper understanding of how H_2_S and GSH are regulated in cancer cells vs. normal cells, and it is also important to identify specific targets that are altered or overexpressed in specific cancer cells. Given that metabolic reprogramming is an established feature of tumor progression, uncovering mechanisms underlying this process may inform the development of therapeutic strategies. Although dietary restriction of methionine or cysteine may be a less toxic alternative to traditional cancer treatments, immune cells (such as T cells, natural killer (NK) cells, and tumor-associated macrophages) also require these essential nutrients for survival, further challenging effective cancer treatments. Additionally, dietary restriction/alteration significantly impacts the gut microbiome and may influence cancer progression by altering host metabolism and immune responses. Thus, it is crucial to identify new targets in the oxidative defense pathway or to develop combination strategies that can effectively eliminate cancer cells and stimulate antitumor immunity.
